# Benchmarking the Base Randomization Algorithm as a Possible Tool for the Initial Step of Generating a Virtual RNA Aptamers Library

**DOI:** 10.3390/biotech14030072

**Published:** 2025-09-12

**Authors:** Kabelo P. Mokgopa, Shina D. Oloniiju, Kevin A. Lobb, Tendamudzimu Tshiwawa

**Affiliations:** 1 Department of Chemistry, Rhodes University, Makhanda 6140, South Africa; k.lobb@ru.ac.za; 2 Department of Mathematics, Rhodes University, Makhanda 6140, South Africa; s.oloniiju@ru.ac.za; 3 Research Unit in Bioinformatics (RUBi), Rhodes University, Makhanda 6140, South Africa

**Keywords:** Aptamers, RNA, Algorithms, Randomization, MFE, virtual library, BRA

## Abstract

While databases are emerging across various domains, from small molecules to genomics and proteins, aptamer databases remain scarce, if not entirely absent. Such databases could serve as a comprehensive resource for advancing research, innovation, and the applications of aptamer technology across multiple fields. This advancement would likely lead to improvements in healthcare, environmental monitoring, and biotechnology. Furthermore, the establishment of aptamer databases would facilitate molecular modelling and machine learning, opening doors to further advancements in understanding and utilizing aptamers. Against this backdrop, in this study, we present and benchmark the Base Randomization Algorithm (BRA) as a potential solution to the scarcity of aptamer databases. Through statistical analysis, we examine key factors such as minimum free energy (MFE), base compositions, and base arrangements. Notably, sequences generated using the BRA exhibit a Gaussian distribution pattern. We also examine the details of how each base within a sequence is chosen using mathematical principles, ensuring that the sequences are valid and optimized statistically. Additionally, we explore how the length of the randomized generated sequences can affect the folding of their structures at both the secondary and tertiary levels. Based on composition analysis, we propose that the base mean of the dataset can be approximated as ${\bar{x}}_{B}\approx P\left(x\right)\;\times \;N$, for dataset of sequences with the same length and ${\bar{x}}_{B}\approx P\left(x\right)\;\times \;M,\;$ where M  is the median and N the mean, for a dataset with randomized length that follows a Gaussian distribution.

## 1. Introduction

For a long time, nucleic acids have been associated with biological functions, including the storage of inherited information (DNA) and gene transfers to protein (RNA) [1,2]. Recent scientific reports have shown that nucleic acids can do much more, including catalysis, detection, and diagnostics [3,4]. Despite these remarkable applications and functions, there are still challenges in exploiting the potential of aptamers; these include dealing with their folding stability. For these reasons, nucleic acid modifications are a strategy for addressing these issues. Although nucleic acids are known to be synthesized biologically, recent studies have resulted in the successful synthesis of non-biological oligonucleotides/nucleic acids using advanced methods such as solid-phase and solution-phase synthesis [5,6,7]. These synthesized nucleic acids are called nucleic acid aptamers. The nucleic acid aptamers are single-stranded nucleic acid oligomers with a high binding affinity towards their targets [7]. The secondary and tertiary structure of an aptamer is responsible for this target affinity and selectivity [8,9]. Due to their high specificity and selectivity, these aptamers are recognized as competitors of antibodies. They can target a wide range of entities, including metal ions, metabolites, proteins, biological cofactors, small molecules and even organisms, such as viruses and bacteria [8,9,10,11].

The possibilities are either RNA aptamers or DNA aptamers, based on their composition, in the same way that biological RNA and DNA are distinguished [12]. RNA aptamers have uracil (U) as one of their nucleotides or bases, while DNA aptamers have tyrosine (T) [13]. It has been demonstrated that DNA aptamers are more stable than RNA aptamers [14,15]. Traditionally, aptamers are generated using SELEX (systematic evolution of ligands by exponential enrichment), which usually takes time [16]. SELEX is an in vitro approach designed to identify and select the aptamer that binds selectively to a target. There are a multitude of SELEX-based methods that have been introduced as modifications of the traditional approaches to increase efficiency and aptamer specificity [7]. These SELEX-modified methods include Counter-SELEX, Cell-SELEX, Capillary Electrophoresis-SELEX, One Step-MonoLEX, Microfluidic-SELEX, and Toggle-SELEX [17,18,19,20,21].

With much experimental research having been conducted, there is still a significant gap in computational work within the aptamer field. One of the reasons for this is that there are limited databases that include aptamer crystal structures or sequences that supply information for simulations; even more recent databases have few aptamer sequences. Although aptamer databases remain limited in scope and accessibility, recent efforts such as AptaCom [22] and related initiatives have begun to address this gap by providing centralized repositories for curated aptamer data. Of course, some progress is apparent in the literature, including the recent SELEX method called “SELEX in-silico”, which allows for a thorough computational exploration of the sequence space [23]. As an alternative approach, in this work, we benchmark a base randomization algorithm by comparing the produced base compositions of RNA aptamers together with sequence folding minimum free energy (MFE) of aptamers from the Aptamer Base database [24]. Based on this benchmarking, we propose this methodology as a potentially neater and faster approach to generating a virtual library of RNA aptamers. Having a virtual library of aptamers then allows for the undertaking of simulations, thereby providing insights into motifs that bind well to the target, together with information relating to the binding process.

## 2. Theory and Methodology

### 2.1. Base Randomization Algorithm

Randomization has been found to be applicable in a variety of fields, such as gaming, sampling, simulations, and art [25]. Algorithms and techniques have been developed to carry out this randomization task, such as Monte Carlo techniques, which are widely used in gaming and computer simulations. Many randomization techniques and algorithms are derived from pseudorandom number generators, which make use of the seed initialization method to produce numbers that appear randomized [26]. The base randomization algorithm presented here makes use of pseudorandom number generation [27]. It generates randomized RNA sequences or “aptamers” where the randomization is both in the bases and in the positions of these bases. The random generation of each single base (or nucleotide) simply follows the following equation:

(1) $$RB=Set\left[{index}_{i}\right]$$

In Equation (1), RB denotes the random base or nucleotide, Set represents a collection of bases, and ${index}_{i}$ is the position of a base in this collection and is calculated according to Equation (2) where the Set is X:

(2) $${index}_{i}=\left\lfloor r\times len\left(X\right)\right\rfloor \;\left\{\begin{array}{c} r\in [0,1)\\ X=\{A,U,G,C\} \end{array}\right.$$

Since the Mersenne twister “random” module from Python 3.9 was used, r is a random float value/number within the half-closed range between 0 and 1 that is generated randomly using the Mersenne twister as the core generator [28]. The pseudorandom Mersenne twister is capable of producing 53-bit precision floats with a period of $2^{19,937}-1$ [29]. X is the Set or collection of elements, which, in this case, are the bases {A, U, G, C} and, in Python scripting, are strings and not numerical values. Since the set X is composed of four strings, then Set can be mapped onto the index set {0, 1, 2, 3} to select the base, and the $len\left(X\right)$ denotes the length of the set X, which is four in this case. It is worth noting that we are dealing with RNA; hence, U (uracil) is present, and T (thymine) is not. The main objective here is to generate multiple sequences of random bases or nucleotides where each individual RNA is unique. A single sequence (seq) may be generated according to Equation (3):

(3) $$\begin{array}{c} seq=\left[{RB}_{0},RB_{1},{RB}_{2},\ldots ,{RB}_{n}\right],\;where\;n\in N_{0},\\ seq=[Set\left[\left\lfloor r_{0}\times len\left(X\right)\right\rfloor \right],\;Set\left[\left\lfloor r_{1}\times len\left(X\right)\right\rfloor \right]\;\ldots Set\left[\left\lfloor r_{n}\times len\left(X\right)\right\rfloor \right]. \end{array}$$

For a single sequence, the generation is based on the sequence length, denoted as n, which is an element of natural numbers (with zero), since we consider the index starting from 0. For multiple lists of sequences with the same length, the sequences ($M_{seqs[]}$) can be expressed as follows:

(4) $$M_{seqs[]}=\left[\begin{array}{ccc} {}[Set\left[\left\lfloor r_{0}^{1}\times len\left(X\right)\right\rfloor \right] & \ldots & Set\left[\left\lfloor r_{n}^{1}\times len\left(X\right)\right\rfloor \right]]\\ \vdots & \ddots & \vdots \\ {}[Set\left[\left\lfloor r_{0}^{m}\times len\left(X\right)\right\rfloor \right] & \ldots & Set\left[\left\lfloor r_{n}^{m}\times len\left(X\right)\right\rfloor \right]] \end{array}\right]=\left[\begin{array}{c} \left[{seq}_{0}\right]\\ \left[{seq}_{1}\right]\\ \vdots \\ {[seq}_{m}] \end{array}\right].$$

For multiple sequences of the same length, $M_{seqs[]}$ is represented as a matrix since it is a list that contains sub-lists of the same length, where the number of sequences m $\in \;N_{0}$. We can thus denote the position of each of these RNA sequences within $M_{seqs[]}\;$ as subscript values of RB. For multiple sequences, which may differ in length, $M_{seqs}$ can also be expressed similarly but with a few additional conditions. Since we are looking at the randomization of sequence length, we denote each sequence as a set rather than as an array, as denoted in Equations (5) and (6):

(5) $$seq=\left\{{RB}_{0},{RB}_{1},{RB}_{2},\ldots ,{RB}_{n}\right\},\;where\;n\in N_{0,}$$

(6) $$seq=\left\{Set\left[\left\lfloor r_{0}\times len\left(X\right)\right\rfloor \right],\;Set\left[\left\lfloor r_{1}\times len\left(X\right)\right\rfloor \right]\;\ldots Set\left[\left\lfloor r_{n}\times len\left(X\right)\right\rfloor \right]\right\},$$

where n is the last position of a base in one of the sequences, automatically indicating that n is the length of that particular sequence. Since this is the case, and the length can be generated randomly between a given closed range, say of j  and k, we can continue and denote it as follows. Let:

(7) $$n\;\;=Set\left[{ind}_{i}\right],\;$$

where:

(8) $${ind}_{i}=\left\lfloor r\times len\left(S\right)\right\rfloor \;\left\{\begin{array}{c} r\in \left[0,1\right)\\ S=\{j,\ldots ,k\} \end{array}\right.\;j,k\in N.$$

Let $M_{seqs}$ be the main set and ${seq}_{i}\;$ be an element: $\left\{{seq}_{i}\right\}$ ∈ $M_{seqs}$; then, for all of ${seq}_{i}\in$ $M_{seqs}$, the main set can be denoted as follows:

(9) $$\begin{array}{cc} M_{seqs} & =\left\{\begin{array}{ccc} \{Set\left[\left\lfloor r_{1}^{1}\times len\left(S\right)\right\rfloor \right] & \ldots & Set\left[\left\lfloor r_{n}^{1}\times len\left(S\right)\right\rfloor \right]\}\\ & \vdots & \\ \{Set\left[\left\lfloor r_{1}^{m}\times len\left(S\right)\right\rfloor \right] & \ldots & Set\left[\left\lfloor r_{n}^{m}\times len\left(S\right)\right\rfloor \right]\} \end{array}\right\}\\ & =\left\{\begin{array}{c} \left\{{seq}_{0}\right\}\\ \left\{{seq}_{1}\right\}\\ \vdots \\ \left\{{seq}_{m}\right\}\\ \left\{{seq}_{m+1}\right\} \end{array}\right\}\;\mathrm{for}\;n\in [i,j] \end{array}$$

For multiple sequences with randomized lengths ($M_{seqs}),$ n is the last position of each sequence in the $M_{seqs}$; therefore, the n values are generated randomly between a closed specified range of j and k. In this study, we choose for n to range between 16 and 60. That said, it is not beyond the realm of possibility that, during the generation of multiple sequences, the algorithm can generate repeating sequences. This concern can be effectively resolved by applying the ‘set’ principle, which enables the creation of a distinct and non-repetitive list of unique items.

The Base Randomization Algorithm (BRA) has a time complexity of O(m×n), where n is the maximum length of the aptamers and m is the number of aptamers to generate. This complexity arises from generating random aptamers, which take O(n) time, and checking for uniqueness using a set that has an average case of O(1). In the worst case, particularly when many attempts are needed to find unique sequences, this could lead to O(m×n) iterations. The space complexity is also O(m×n) due to the storage of unique aptamers in the set. Ultimately, both time and space complexities reflect the efficiency and potential challenges of generating a specified number of unique aptamers.

The number of possible sequences that can be generated using BRA is determined by length n and k number of different bases given, and the formula is defined as:

(10) $$\mathrm{Number}\;\mathrm{of}\;\mathrm{sequences}=k^{n}$$

For sequences composed of four nucleotides (‘A’, ‘C’, ‘U’, and ‘G’), the number of possible sequences is calculated by reducing k to 4. To illustrate how the number of possible sequences increases with sequence length, consider the following calculations. For a sequence of length 1, there are $4^{1}=4$ possible sequences. For a sequence of length 5, the number of sequences increases to $4^{5}=1024$. When the sequence length is extended to 10, the number of possible sequences becomes $4^{10}=10,485,764$. This demonstrates how exponentially the number of possible sequences increases with sequence length, reflecting the vast complexity and variability possible in nucleotide sequences. This exponential growth reflects the combinatorial complexity of variations in nucleotide sequences, indicating that longer sequences can encode a vastly greater number of potential configurations. As the sequence length increases, the number of possible distinct aptamer sequences expands rapidly, providing a larger space for genetic or chemical diversity. This rapid increase in the possible aptamer sequences that can be obtained highlights the richness of the chemical space available. BRA offers a transparent, controllable way to explore the nucleotide composition space.

### 2.2. Generation of Aptamers Sequences

Three sets of aptamer sequences are explored here. Aptamers sequences were generated using a “Base Randomization Algorithm” with Algorithm 1 written in Python. Two lists of aptamers were generated. The first list contained 1100 aptamer sequences with a fixed length ($M_{seqs[]}$) of 22 nt (nucleotides) and the second list contained 20,000 aptamer sequences with a randomized length ranging between 16 and 60 nt ($M_{seqs}$). The third dataset was obtained from an aptamer base, and, in this study, the dataset is referred to as “RNAbase” [29]. This RNAbase dataset contained random RNA and DNA aptamer sequences, which are obtained from experimental work, together with their properties. The DNA sequences were filtered out, and only 904 RNA sequences were left and taken further for composition and structural analysis.
**Algorithm 1: Base Randomization Algorithm (BRA)****Input:** - length: the length of the aptamers to generate (either a specific length or “randomize”) - aptamers numbers: the number of aptamers to generate **Output:** - A list of unique aptamers based on the aptamer number input **Steps:** 1. seed (0) 2. Initialize an empty set() aptamers to avoid the repeats in list 3. If length is “randomize”, then:          a. While the size of aptamers is less than aptamers numbers:                        i. Generate a random length number between 16 and 60 (inclusive)                       ii. Generate a random aptamer as an item using characters ‘ACUG’                       iii. If the aptamer sequence is not in aptamers, then add it to aptamers 4. If length is a specific value, then:            a. Generate a random aptamer of the specified length using characters ‘ACUG’            b. While the size of aptamers is less than aptamers numbers:                     i. Generate a random aptamer of the specified length using characters ‘ACUG’                     ii. If the aptamer is not in aptamers, add it to aptamers 5. Convert the set aptamers to a list and return the list

### 2.3. Secondary and Tertiary Structure Prediction

Single-stranded RNAs fold within themselves through base pairing, resulting in stable secondary hairpin structures. To address the concern of base pairing regions in RNA molecules, RNA folding is essential to map the possible base pairing regions that can be conserved within the molecule. In order to fold a biological molecule computationally, certain tools are required, such as RNAfold [30] and Mfold [31]. For this current study, secondary structures were predicted using the in-house tool named T_SELEX, a program [32] that makes use of the RNAfold algorithm as developed by (Mathews et al.) [30]. The RNAfold algorithm makes use of the Zuker and Steigler algorithm and John McCaskill’s algorithm of partition function [32]. On that note, Zuker and Steigler’s algorithm in RNAfold enables the prediction of minimum free energy (MFE) structures from just a simple given RNA sequence [33]. Here, for 3D (tertiary structure) prediction, all the sequences together with their secondary structures (as predicted using RNAfold) in the previously described datasets were submitted to RNAComposer [34].

## 3. Results and Discussion

### 3.1. Us, Gs, Cs and Us Composition Analysis

**Figure 1:**
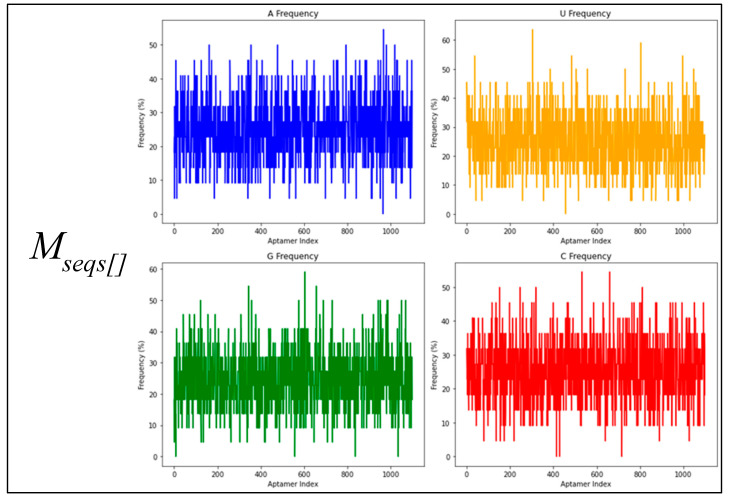

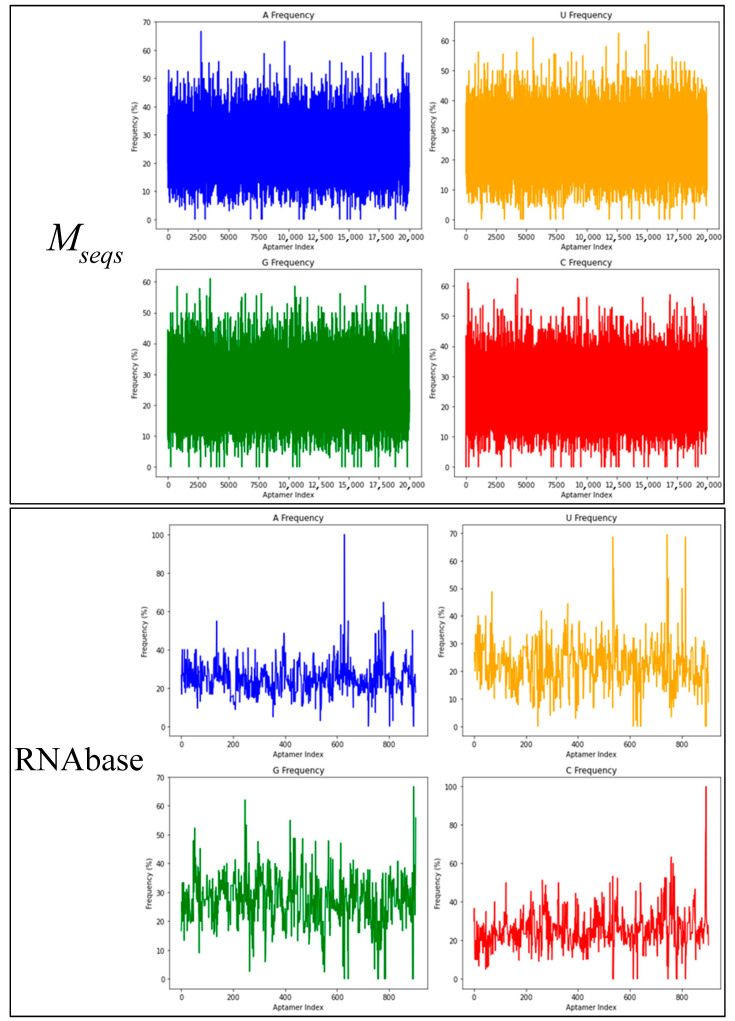
Individual base composition noise plots of the datasets $M_{seqs[]}$, $M_{seqs}$ and RNAbase.

This section focuses on unravelling the base composition of aptamer sequences in the three datasets (fixed length, randomized length, and the set from RNAbase). The single-base compositions of the three datasets ($M_{seqs[]}$, $M_{seqs}$, and RNA base) are compared in Figure 1. The $M_{seqs[]}$ dataset was generated using BRA with a fixed aptamer length of 22 nt, the $M_{seqs}$ dataset was generated using BRA with an aptamer length ranging between 16 and 60 nt, and the RNA base is composed of RNA sequences from aptamer bases [28].

In the study of base composition across different datasets, the behavior of nucleotides in terms of randomness and noise was analyzed, as shown in Figure 1. This figure presents four frequency plots in percentages for each dataset, focusing on specific RNA aptamers. The *x*-axis numerically labels the aptamers (e.g., “aptamer 1,” “aptamer 2,” …, “aptamer 1100”), which are referred to as the aptamer index; this helps track individual sequences. Each plot corresponds to one of the nucleotides: Uracil (U), Guanine (G), Adenine (A), or Cytosine (C).

Figure 1, which represents the BRA datasets, reveals that most aptamers in $M_{seqs[]}$ have nucleotide frequencies ranging between 15% and 35% for each base, while, in the $M_{seqs}$ dataset, the frequency ranges from 10% to 45%. Many aptamers contain similar amounts of each nucleotide across these two aptamer datasets. For RNAbase, the data are not clear enough to draw definitive conclusions about the nucleotide composition frequency of most aptamers. Across all datasets, including RNAbase, some aptamers completely lack certain bases. This is observed within all frequency noise plots, with some aptamers having a nucleotide frequency of 0%. This indicates that some aptamers may be synthesized without uracil, guanine, cytosine, or adenine, whether intentionally or unintentionally. Additionally, of course, with the BRA datasets ($M_{seqs}$ and $M_{seqs[]}$), it was not intentional; nevertheless, this pattern of missing nucleotides was observed. The absence of guanine (G) in some sequences within the three datasets is particularly concerning, as G-rich sequences are known to form stable secondary structures. When examining the RNAbase dataset (Figure 1), unusual trends appear, with some aptamers containing 100% of a single nucleotide. This indicates that these aptamers cannot form stable folded structures due to the lack of complementary nucleotides for pairing. This underscores the importance of thoroughly investigating base composition in relation to RNA folding.

Table 1 presents the summary statistics of nucleotide frequencies (U, G, A, C) across three datasets. In both the Mseqs and Mseq22 datasets, the average number of occurrences for each nucleotide is approximately equal: around 5 in $M_{seqs[]}$ and 9.35 in $M_{seqs}$ with small variances. This indicates that the base randomization algorithm was successful in producing balanced and unbiased nucleotide distributions. In contrast, the RNAbase dataset shows marked differences in both mean and variance among nucleotides. Guanine (G) appears more frequently on average (15.12) compared to uracil (U), adenine (A), and cytosine (C), and its variance is also higher. This suggests that real RNA aptamers may evolve with inherent sequence preferences or structural constraints that favor certain bases over others.

To test whether the differences in nucleotide means were statistically significant, a one-way ANOVA was conducted for each dataset, as shown in Table 2. The ANOVA results for $M_{seqs}$ and $M_{seqs[]}$ yielded *p*-values of 0.875 and 0.429, respectively, both of which are well above the standard alpha level of 0.05. The F-statistics in both cases were also below the corresponding F-critical values. These results confirm that, in the randomized datasets, there are no significant differences among the base means, validating the effectiveness of the base randomization algorithm. However, for the RNAbase dataset, the ANOVA yielded a highly significant *p*-value (*p* < 0.001) and an F-statistic well above the critical value, indicating that the nucleotide frequencies differ significantly. This statistical evidence supports the observation that real aptamers do not follow a uniform distribution of nucleotide bases and instead exhibit sequence biases that may relate to structural or functional constraints.

**Figure 2:**
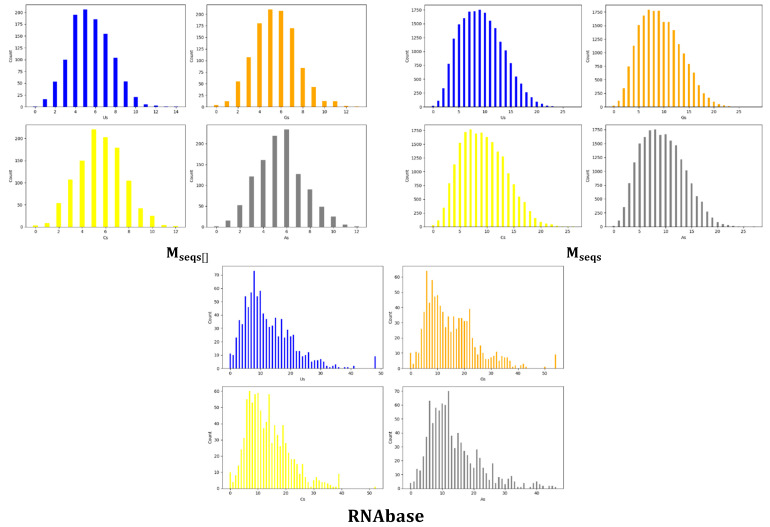
Composite of individual base distribution plots within the datasets $M_{seqs[]}$*,* $M_{seqs}$ and ***RNAbase***.

The distribution count plots in Figure 2 show how often each nucleotide appears in sequences within each dataset. In our study, the focus is on counting Uracil (U), Adenine (A), Guanine (G), and Cytosine (C) in various aptamer sequences across three datasets. The initial noise plots for $M_{seqs[]}$ suggested that most aptamers had a nucleotide frequency ranging between 15% and 35%, while, for $M_{seqs}$, most aptamers exhibited a single nucleotide frequency ranging from 10% to 45%. However, Figure 2 provides more clarity, showing that, in the $M_{seqs[]}$ dataset, most aptamers have base composition within a sequence ranging from 4 to 8 nt, while, in the $M_{seqs}$ dataset, counts vary from 3 to 20 nt. For the *RNAbase* dataset, pinpointing a specific range is trickier due to its multimodal distribution. Nevertheless, it appears that many aptamer sequences in *RNAbase* have base counts between 5 and 20 nt, although this is not consistent across all four bases, as illustrated in Figure 2. The distribution plots indicate that the $M_{seqs[]}\;$ dataset base counts follow a normal distribution, whereas the *M_seq_* dataset shows a slight leftward skew. This skew is likely due to the random lengths ranging from 16 to 60 nt. Figure 2 (for these two datasets) reveals a similar distribution for all four bases, indicating that they have comparable mean values. We confirmed this through a one-way ANOVA, shown in Table 1.

Based on Figure 2, we initially assumed that the nucleotide frequency distributions in the $M_{seqs[]}$ dataset followed a normal distribution. To statistically test this assumption, we applied both the Shapiro–Wilk and Anderson–Darling normality tests to each base across all three datasets (Table 3). For $M_{seqs[]}$, all bases (U, G, A, and C) returned Shapiro–Wilk *p*-values of 0.0000 and Anderson–Darling statistics well above critical values, indicating significant deviation from normality despite the distributions appearing symmetric or bell-shaped visually. This highlights the limitations of visual inspection and reinforces the need for formal testing. Similarly, the $M_{seqs}$ dataset, which is much larger (*n* = 20,000), also failed both normality tests across all bases, with even more extreme Anderson–Darling values. These results suggest that, due to large sample sizes, even minor deviations from normality are detected with high statistical power. In the case of ***RNAbase***, all four nucleotide distributions also rejected normality under both tests, with the lowest Shapiro–Wilk statistics among all datasets. This confirms that real aptamer base distributions not only deviate from uniformity but also do not follow a Gaussian distribution, further emphasizing their inherent sequence biases. Consequently, parametric tests assuming normality may be inappropriate for such datasets unless justified with transformation or robust alternatives.

As shown in Table 4, Kolmogorov–Smirnov (K–S) tests were performed to assess whether the distributions of base frequencies (U, G, A, C) differ significantly across the three datasets. All pairwise comparisons produced D-statistics ranging from 0.22 to 0.65, with *p*-values of approximately zero, suggesting statistically significant differences in the distributions for every base across all dataset pairs. These results confirm that BRA datasets yield markedly distinct base frequency profiles when compared to the *RNAbase* dataset. Such differences affirm that base randomization significantly alters the nucleotide composition landscape, thus validating the variability introduced by our algorithm.

### 3.2. Adjacent Base Composition

**Figure 3:**
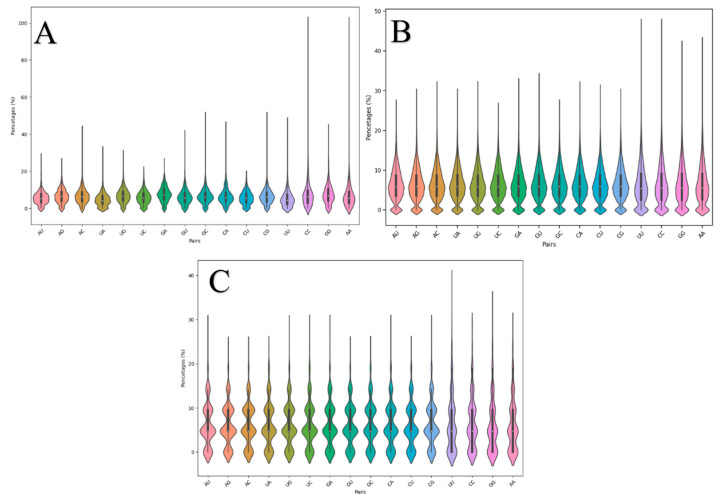
Composite figure of violin plots for datasets (**A**)–(**C**), where (**A**) is composed of adjacent base composition distribution plots within the dataset $M_{seqs[]}$, (**B**) for $M_{seqs}$ and (**C**) for ***RNAbase***.

The violin plots in Figure 3 show the distribution of the adjacent base compositions. Like the individual base compositions, the distributions for adjacent base pairs are mostly similar for most pairs within each dataset, as seen in Figure 3A–C. Although a difference was expected in terms of the distributions within the adjacent base composition of the RNAbase dataset, unfortunately, it was not observed, as shown in Figure 3C. This expectation of variation was based on fact that the RNAbase (C) dataset contained aptamer sequences from experimental SELEX studies, while, for BRA, the dataset sequences (A and B) were generated theoretically [31]. Figure 3B shows that adjacent base pairs from AU to CG have similar median values and quartiles, with some differences from UU to AA (which are the last four violin plots in Figure 3B). The highest probabilities for these pairs correspond with their median values. The same is observed in Figure 3A, where adjacent pairs from AU to CG also share the same minimum, first quartile, median, third quartile, and maximum values. The median is close to the first quartile, indicating a slight positive skew in the data. The most common value peaks around a median of 5, with smaller peaks at 0 and 10. The presence of a minor peak at zero suggests that some aptamers do not have certain adjacent base compositions. All adjacent pair compositions have a minimum value of zero, meaning some aptamers do not include those pairs at all. The distributions from UU to AA show similar trends, although with minor differences. The BRA algorithm rarely places identical bases next to each other but does not completely rule it out. Median values are consistent across the AU to AA range. Figure 2B shows uniform distributions from AU to CG, with slight variations in the UU to AA range. Interestingly, a similar pattern is observed in Figure 2C, with adjacent base pairs from AU to AA having the same minimum, first quartile, median, third quartile, and maximum values. This shows that, although RNAbase exhibits a multimodal distribution of the adjacent base pair compositions, the pattern is also similar to that of the BRA algorithm, even though the datasets do not have the same size.

### 3.3. Folding, Secondary Structure, and 3D Predictions

**Figure 4:**
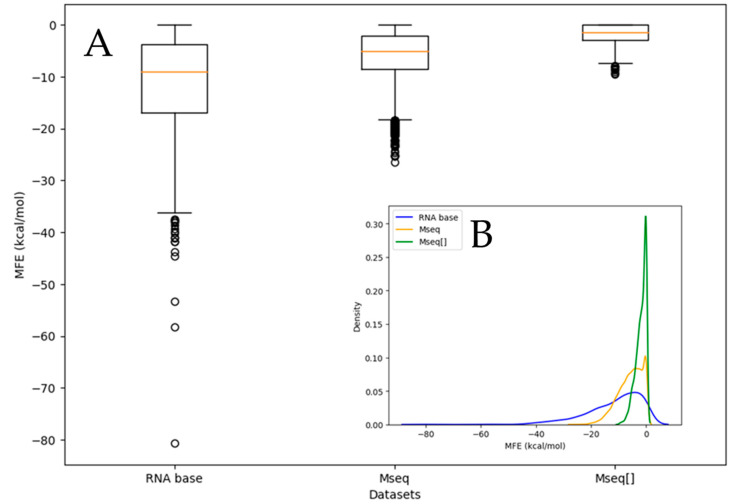
Composite figure of A and B, where (**A**) is composed of box plots of the MFE of RNA aptamers within the datasets $M_{seqs[]}$, $M_{seqs}$, and ***RNAbase***. (**B**) shows the distribution line plots of the RNA aptamers within the datasets $M_{seqs[]}$, $M_{seqs}$ and ***RNAbase***.

Figure 4A shows the distribution of the minimum free energy (MFE) values for aptamer sequences across the datasets we investigated. The analysis revealed that RNAbase contains the most stable aptamers, with the most stable one reaching an MFE of −80.70 kcal/mol. This trend is further illustrated by the outliers in the box-and-whisker plot for RNAbase in Figure 4A. Other highly stable aptamers in this dataset have MFEs of −58.00 kcal/mol and −53.29 kcal/mol, along with a notable number of outliers between −46 kcal/mol and −37 kcal/mol. The significant difference between the most and second most stable aptamers suggests that sequence length contributes to variations in MFE. The low MFE of RNAbase aptamers suggests that these aptamers are likely longer, as MFE generally decreases with more base pairings, indicating greater stability. Notably, 61 aptamers in RNAbase have an MFE of zero, accounting for 6.75% of the dataset, which could be due to the relatively short sequences in RNAbase, since aptamer sequence length within this dataset ranges from 3 to 180 nt. Although typical aptamer lengths cited in the literature are between 16 and 60 nt, RNAbase includes many shorter sequences, which may not fold into stable structures but could provide more binding surface area for targets. While synthesizing very short nucleic acids can be tricky, they still have practical applications [29].

In the $M_{seqs}$ dataset, the most stable aptamer has an MFE of −26.39 kcal/mol (aptamerd5165) and a length of 54 nt. Although this aptamer is stable, the maximum length in this dataset is 60 nt, indicating that, while length is a factor influencing MFE, it is not the only factor. Other factors, such as base composition (both individually and in pairs) and base positioning, also significantly impact stability. Figure 4A,B show that aptamer 18670, which is 50 nt long, has an MFE of −25.39 kcal/mol, followed by other aptamers with slightly higher MFEs. Out of 20,000 aptamers in the $M_{seqs}$ dataset 1942 have a MFE of zero, suggesting that 9.71% do not fold.

Further examination of the $M_{seqs[]}$ dataset reveals that the most stable aptamer has an MFE of −9.5 kcal/mol (aptamer1084). Since all aptamers in this subset are 22 nt long, length certainly does not influence the distribution of MFE in this case. The second most stable aptamer has an MFE of −9.3 kcal/mol (aptamer950). Among these 1100 aptamers, 281 have an MFE of zero, meaning that 25% do not fold. Overall, $M_{seqs[]}$ displays the highest percentage of non-folding aptamers, confirming that, while length affects MFE, it is not the sole factor to consider.

**Figure 5:**
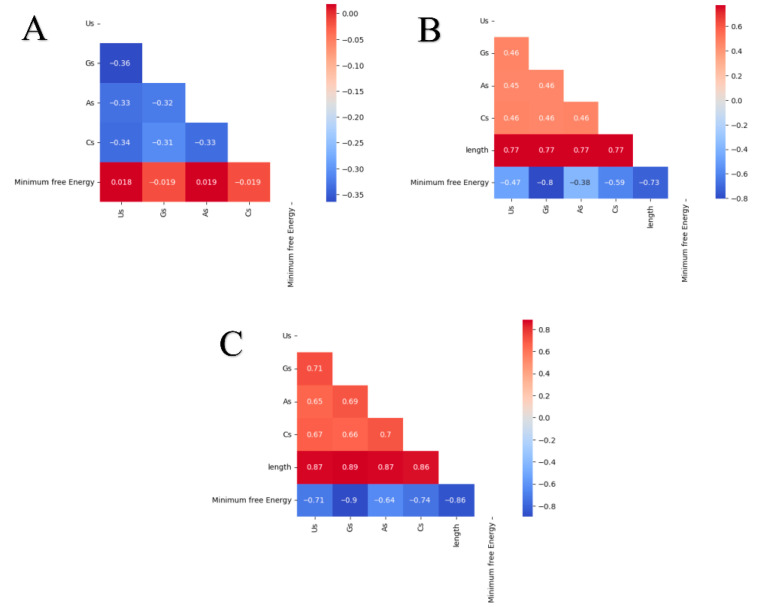
Correlation matrices of bases, length of the sequences and the minimum free energy of the three datasets, where (**A**) is for a dataset $M_{seqs[]}$, (**B**) for $M_{seqs}$ and (**C**) for ***RNAbase***.

Correlation heatmaps were constructed to evaluate and investigate the correlation between the length of each sequence in each dataset and their folding behavior through observing MFE, as shown in Figure 5B,C. Before discussing the folding and correlations in detail, it is important not to overlook composition correlations. For the $M_{seqs[]}\;$ dataset, their correlation is reported in Figure 5A. There is a distinctive correlation of −0.34 to −0.32 among the individual base compositions in the $M_{seqs[]}\;$ dataset. This suggests that there is an inverse relationship among the bases, even though the correlation is not strong enough. This could be because the sequences have the same length, and, if one base were to dominate, the other bases would have to be reduced, thereby ensuring that the combined total remains at 22 nt. For instance, if the number of As in a sequence is 10, then other bases will have to share the remaining twelve compositions to make it up to 22, hence the negative correlation. On the contrary, the bases for the $M_{seqs}$ and RNAbase datasets show a positive correlation, which suggests that randomized length has a significant positive relationship that can be observed among the bases.

Figure 5B shows a relative strong correlation of −0.73 between length and MFE. Notably, in the RNAbase dataset, this correlation is even stronger at −0.9. This indicates a significant inverse relationship between the stability of RNA molecules and their length. While correlation does not imply causation, these results suggest that length plays a substantial role in RNA folding stability. The inverse relationship emphasizes the idea that longer RNA sequences tend to have lower MFE values, implying greater stability. This occurs because longer sequences have more plausible ways of folding through base interactions, which can contribute to more stable structures. However, it is important to note that not all possible folding states are stable. Thus, understanding the stability of RNA involves more than just length. It also requires an analysis of the composition and positioning of individual bases within the sequence. This highlights the importance of considering both base composition and spatial arrangement in relation to the overall stability of RNA molecules.

**Figure 6:**
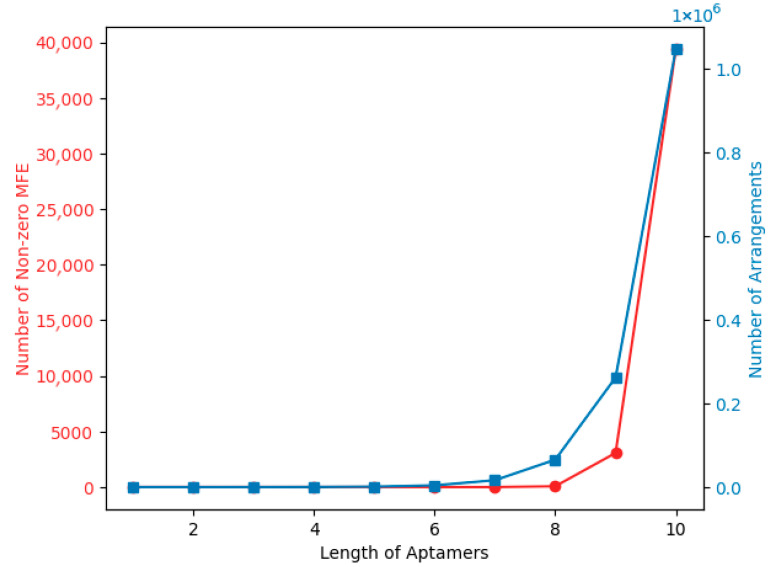
Analysis of the number of possible base rearrangements (blue) and the number of possible folded aptamers or non-zero MFE aptamers as the length increases using BRA.

Because aptamer length, composition, and position or arrangement influence the aptamer MFE, it can also be thought of as a product of a length-dependent factor, aptamer length, composition, and the arrangement of nucleotides. According to Trotta’s work, if MFE = a + b × length, then MFE/length = a/length + b in the case of perfect linear relationship between MFE and length [35]. However, Trotta further demonstrates that the assumption of a linear relationship between length and MFE is invalid [35]. This further justifies that there is more that needs to be taken into consideration about the composition and arrangement of nucleotides towards MFE. Although there is not a clear path towards formulating exactly how composition and arrangement affect MFE, in order to give a clear and probable hypothesis, we can assume that these factors do contribute to it. Given that folding will not occur if the base length is less than or equal to 7, we can denote our hypothesis as $MFE=-\left(ɀ\;f\;N\right),$ where ɀ=0 if N≤7 and ɀ=1 if N>7. N is the length of the sequence and f represents both composition and arrangement factors, even though we cannot yet give a precise equation for how f may be calculated. ɀ is the length-dependent factor, which is introduced based on the understanding that all sequences that have any length less than or equal to 7 have MFE = 0. This suggests that the arrangement does not matter in that case: the composition and arrangement of nucleotides matter only if the *N* is greater than 7. The calculations to back up this claim about ɀ are provided in Figure 6.

The graph in Figure 6 shows the exponential relationship between aptamer length and two factors: the number of stable structures with nonzero minimum free energy and the number of possible arrangements. As aptamer length increases, there is a significant rise in the number of stable structures with a nonzero MFE, suggesting that longer aptamers are more likely to form stable structures. It is important to note that the red line indicates a sharp increase in the “Number of nonzero MFE” after the length of aptamers reaches 8. This could be because longer sequences have more potential for forming stable secondary structures [36,37,38,39].

According to Zuker’s algorithm, a permissible secondary structure must have a loop that has three free nucleotides and two base pairs [40,41]. This is because a loop with fewer than three free nucleotides would be too tight to form, and a base pair contributes significantly to the stability of the structure. Moreover, in sequences shorter than 8, there might not be enough nucleotides to form these stable structures with the required loop and base pairs. Hence, a sharp increase is observed in the number of nonzero MFEs after length 7, suggesting that longer sequences have more potential for forming these stable structures [42]. Additionally, the number of possible arrangements increases with aptamer length, attributing to the exponential increase, indicating a saturation in arrangement diversity. Overall, the trend illustrates the complexity and diversity of aptamer interactions, with longer aptamers having a higher potential for stable structures and arrangement variety.

**Figure 7:**
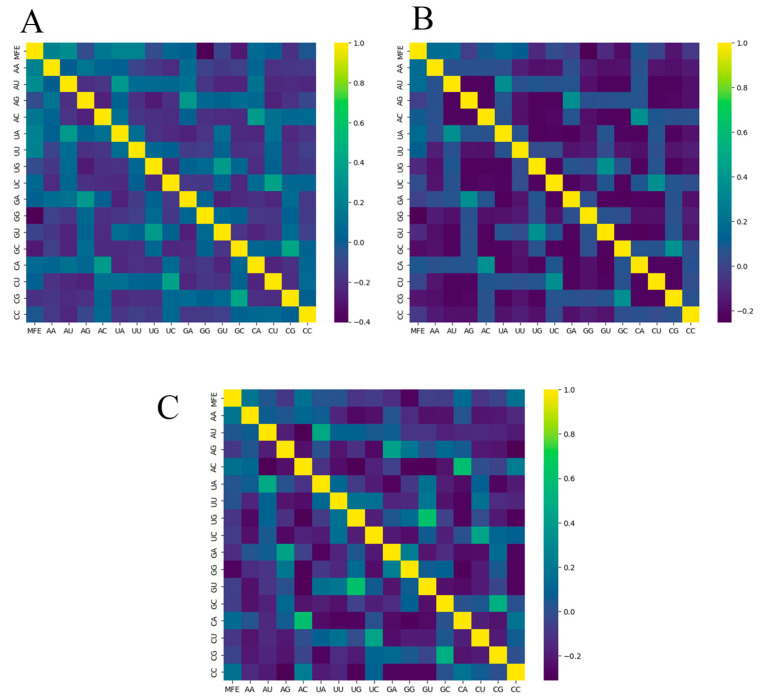
Correlation matrices of adjacent base composition within a sequence and the minimum free energy (MFE) of the three datasets, where (**A**) is for a dataset $M_{seqs[]}$, (**B**) isfor $M_{seqs}$, and (**C**) is for ***RNAbase***.

To gain insights into how adjacent base compositions influence the minimum free energy (MFE) and thereby contribute to RNA molecule stability, we examined the heatmaps given in Figure 7. Notably, GG adjacent base compositions exhibit a consistent, but still not significant, negative correlation with the MFE across all datasets. Intriguingly, other adjacent base compositions such as GC, CG, GU, and AG also display a slight negative correlation with the MFE in all datasets. This suggests that sequences containing GG may favor folding, especially if UU, CC, UC, or CU exist in the sequence. A similar assumption can be made for the other mentioned pair compositions. Despite variations in correlation values, the heatmaps exhibit similar patterns across all three datasets, indicating consistent trends from AA to UA on the *y*-axis and from GC to CC. However, overall, there is not a strong relationship observed between adjacent base composition and MFE.

Table 5 shows the two best-folded aptamers from each dataset; this is presented in terms of sequences (together with pseudoknots), MFE secondary structures, and tertiary structures. Regarding the motifs found in these RNAbase aptamers, RNAse69 has a four-way junction or a multi-dimensional loop in the center, suggesting a complex structure that may enhance its binding capabilities [43]. In contrast, RNAse192 features a simpler dodecahedral structure with kinks but lacks a multi-dimensional loop. Despite both sequences being long, they have different secondary structures. For the $M_{seqs}$ aptamers, Aptamer5165 displays a secondary structure that includes a multi-center loop, indicating potential for varied interactions. On the other hand, Aptamer18670 forms a more straightforward structure with good stem-loops and internal loops, but no kinks, suggesting a simpler binding profile [44,45]. Finally, $M_{seqs[]}$, Aptamer960 and Aptamer1084 show simple secondary structures with no multi-center loops. This likely means their shorter sequences do not have the length needed to form more complicated, stable structures. It is important to highlight that BRA serves as the foundational algorithm for the T_SELEX [31] tool, a Python-based tool currently under review, which enables the large-scale generation of RNA aptamer libraries along with secondary and tertiary structure prediction and docking integration using tools such as RNAComposer [33].

### 3.4. PCA and t-SNE Nucleic and Chemical Space

The dimensionality reduction analyses were performed on a comprehensive set of aptamer features that characterize their sequence, structure, and thermodynamics. These features include nucleotide composition percentages (A, U, G, C), dinucleotide and k-mer frequencies (up to k = 3), and adjacency patterns of nucleotides, which are used to capture sequence and structural motifs such as loops and pairings (total features: 161). Structural attributes derived from predicted secondary structures, such as the number of paired and unpaired bases, stem density, and GC/AU skew, were incorporated to represent folding properties. Additionally, thermodynamic features such as melting temperature and Shannon entropy were included to describe stability and sequence complexity. All numeric features were normalized before applying PCA and t-SNE to ensure comparability. This rich feature set enabled an informative projection of the aptamers’ chemical space, reflecting both their compositional diversity and biophysical properties.

**Figure 8:**
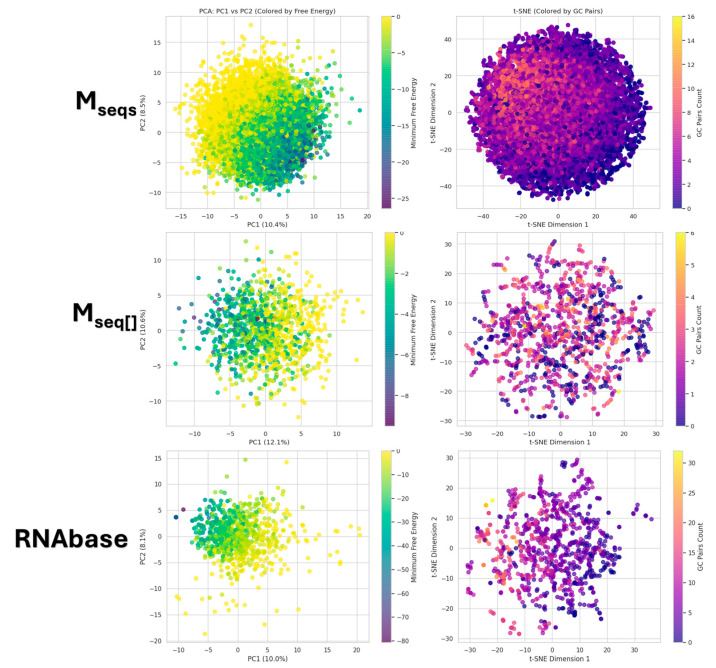
Principal component analysis (PCA) and t-distributed stochastic neighbor embedding (t-SNE) plots of datasets $M_{seqs[]}$, $M_{seqs}$ and RNAbase.

Figure 8 presents a comprehensive visualization of the chemical space of aptamers through dimensionality reduction techniques. On the left, principal component analysis (PCA) scatter plots illustrate the distribution of aptamers across the first two principal components (PC1 and PC2), which together explain approximately 18–23% of the variance in the data. The points are colored based on their minimum free energy (MFE), highlighting how energetic stability varies across the chemical space. On the right, t-distributed stochastic neighbor embedding (t-SNE) plots provide a nonlinear mapping of the data, with points colored by the count of GC base pairs. These visualizations reveal underlying patterns in the aptamer population, where clusters or gradients emerge based on structural stability (MFE) and pairing patterns.

In our analysis of the dimensionality reduction plots, the PCA visualizations revealed distinct spatial patterns between the datasets. For the $M_{seqs}$ dataset, the more stable aptamers (green, indicating lower MFE values) were predominantly located on the right-hand side of the PCA plot, while less stable aptamers (yellow, indicating higher MFE values) clustered on the opposite side. In contrast, for the other datasets, the more stable aptamers appeared on the left-hand side. The t-SNE plots further highlighted dataset-specific structural organization: in both datasets, aptamers tended to form a compact, ball-like distribution, with significant aptamers showing higher GC pairings occupying notable regions within this structure. For $M_{seqs}$, aptamers with greater pairing density were concentrated toward the center-left of the ball, whereas, in the other dataset, these highly paired sequences were more evenly distributed throughout the t-SNE space. Notably, PCA captures global variance trends, while t-SNE uncovers local clustering reflecting sequence motifs and structural features.

## 4. Remarks and Propositions

Throughout the course of the study, various remarks and propositions emerged, which were identified as promising areas for further exploration. These noteworthy aspects, along with their corresponding mathematical proofs, are documented below. Using the two BRA-generated datasets, we propose that the mean composition of each base in each dataset can be approximated as follows:

For *N* fixed length of sequences:

(11) $${\bar{x}}_{B}\approx P\left(x\right)\times N\;x\;\epsilon \;X\;where\;X=\left\{A,U,G,C\right\}$$

Since $P\left(x\right)=\frac{1}{n},\;$ for n being the lengh of set X, then :

(12) $${\bar{x}}_{B}\approx \frac{1}{n}\times N$$

For *N* as random length between closed range of i,j; where i≠j , then:

(13) $${\bar{x}}_{B}\approx \frac{1}{n}\times M\;where\;M\;\mathrm{is}\;\mathrm{median}\;\mathrm{value}\;\mathrm{of}\;[i,j]$$

**Proofs.**:

1. Statement, Equation (11)

Claim: A base x mean of a dataset can be approximated as the product of the probability of base x in set X and the length of the sequences, given that all sequences in the dataset have the same length:

$${\bar{x}}_{B}\approx P\left(x\right)\times N$$

**Proof** **1.** For each sequence seq whose length is N, for any x ϵ X={A,U,G,C}:

$$\frac{k}{N}\approx P\left(x\right)$$

if N  is large enough and if it follows the Gaussian distribution, and where 𝓀 is the number of x  in sequences, *seq*, and *P(x)* represents the probability of randomly selecting base x. Therefore, 𝓀 can be explicitly approximated as:

$$k=P\left(x\right)\times N$$

Since the length of the sequence is the same, we expect the count of base X in each sequence to be approximately the same. Then, we can average the number of occurrences of the base x as:

$${\bar{x}}_{B}=\frac{1}{M}\sum_{i=1}^{M}k_{i}\approx \frac{Mk}{M}=P\left(x\right)\times N$$

Note: $k_{1}\approx k_{2}\;\approx k_{3}\;\ldots \;\approx k_{M}$

2. Statement Equation (13)

Claim: A base x mean of a dataset set can be approximated as the product of the probability of base x in set X and the mean or median of lengths of the sequences, given that sequences in the dataset have the random lengths of closed range i and j.

$${\bar{x}}_{B}=\frac{1}{n}\times M_{□}$$

**Proof** **2.** For m sequences:

Assumption:

Each sequence has different length:

$$N_{1},N_{2}N_{3}{,\ldots ,N}_{M}$$

For large enough $N_{1},N_{2}N_{3}{,\ldots ,N}_{M}$, then:

$$\frac{k_{1}}{N_{1}}\approx P\left(x\right),\frac{k_{2}}{N_{2}}\approx P\left(x\right),\frac{k_{3}}{N_{3}}\approx P\left(x\right),\ldots ,\frac{k_{M}}{N_{M}}\approx P(x)$$

The counts:

$$k_{1}\approx P\left(x\right)\times N_{1},\;k_{2}\approx P\left(x\right)\times N_{2},\;k_{3}\approx P\left(x\right)\times N_{3},\;\ldots ,k_{m}\approx P\left(x\right)\times N_{m}$$

Average number of occurrences:

$$\begin{array}{c} {\bar{x}}_{B}=\frac{1}{M}\sum_{i=1}^{M}k_{i}=\frac{k_{1}+k_{2}+k_{3}+\ldots +k_{M}}{M}\\ =\frac{\left(P\left(x\right)\times N_{1}\right)+\left(P\left(x\right)\times N_{2}\right)+\left(P\left(x\right)\times N_{3}\right)+\ldots +\left(P\left(x\right)\times N_{m}\right)}{M}\\ =P(x)\frac{\left(N_{1}+N_{2}+N_{3}+\ldots +N_{m}\right)}{M} \end{array}$$

Since we know that, for uniform distribution, mean is equal to median, then:

$$\bar{x_{B}}\approx P\left(x\right)\times M$$

For $P\left(x\right)$= $\frac{1}{n}$:

$$\bar{x_{B}}\approx \frac{1}{n}\times M_{□}$$

These proofs lay a solid background for understanding the variance and covariance of bases in aptamer libraries. This further validates the assumption that base distributions in BRA-generated sequences are not arbitrary but follow probabilistic expectations. By examining the expected distribution of bases, researchers can assess the diversity of sequences, which is essential for effective target binding. The average occurrences of bases help predict how often they appear in random sequences, guiding the design of experiments and the generation of libraries.

## 5. Conclusions

In conclusion, the analysis revealed diverse base compositions in RNA aptamers, with implications for stability based on the presence or absence of specific nucleotides. The study emphasizes the importance of understanding base pairings and compositions for predicting the stability of RNA structures. Through benchmarking BRA, we provide a mathematical aspect of how this algorithm works to generate sequences, whereby multiple sequences of the same length ($M_{seqs[]}$) can be denoted as matrices; meanwhile, sequences with random lengths (and $M_{seqs}$) can be thought of as the main set and subsets. The compositions and arrangement, together with the MFE of the generated sequences in the $M_{seqs[]}$ and $M_{seqs}$ datasets, were evaluated and compared to the RNA aptamer sequences from Aptamerbase (RNAbase). The results of the one-way ANOVA tests across all datasets ($M_{seqs[]}$, $M_{seqs}$ and *RNAbase*) indicate no statistically significant differences in the mean counts of nucleotide bases (A, U, G, C), suggesting that base distribution is relatively uniform within each dataset. However, normality tests (Shapiro–Wilk and Anderson–Darling) and KS-based pairwise comparisons reveal significant deviations from normality and statistically significant distributional differences between datasets, particularly highlighting the distinct nature of base patterns in synthetic BRA dataset versus experimental (RNAbase) sequences. Based on the composition analysis, we propose that the base mean of the dataset can be approximated as ${\bar{x}}_{B}\approx P\left(x\right)\;\times \;N$, for a dataset of sequences with the same length and ${\bar{x}}_{B}\approx P\left(x\right)\;\times \;M$ for a dataset with randomized lengths that follow a Gaussian distribution. Finally, we discuss and highlight an important aspect regarding the folding of aptamers generated by the BRA algorithm. Specifically, it is noted that aptamers with lengths equal to or less than 7 nt lack the ability to fold when utilizing RNAfold. This emphasizes that aptamers with longer length are more likely to exhibit very low MFE values, suggesting very stable folded aptamers.

## Figures and Tables

**Table 1:** One-way ANOVA summary of base composition across U, G, A, and C.

Dataset	Base	Count	Sum	Average	Variance
$M_{seqs}$	U	20,000	186,997	9.3498	17.0529
	G	20,000	187,102	9.3551	17.0290
	A	20,000	187,605	9.3802	17.1471
	C	20,000	187,053	9.3526	17.3570
$M_{seqs[]}$	U	1100	6073	5.5209	4.2371
	G	1100	6003	5.4573	4.0173
	A	1100	5994	5.4491	3.9728
	C	1100	6130	5.5727	3.9119
**RNAbase**	U	904	11,292	12.4912	67.5304
	G	904	13,669	15.1206	90.6200
	A	904	12,327	13.6361	67.9062
	C	904	12,317	13.6250	59.3465

**Table 2:** ANOVA results table.

Dataset	Source of Variation	SS	df	MS	F	*p*-Value	F Crit	Significance (α = 0.05)
$M_{seqs}$	Between Groups	11.180	3	3.933	0.229	0.876	2.605	No
	Within Groups	1.37 × 10^6^	79,996	17.146				
	Total	1.37 × 10^6^	79,999					
$M_{seqs[]}$	Between Groups	11.158	3	3.719	0.922	0.429	2.607	No
	Within Groups	17,736.842	4396	4.035				
	Total	17,748.000	4399					
**RNAbase**	Between Groups	3152.917	3	1050.972	14.730	0.000	2.607	Yes
	Within Groups	257,718.926	3612	71.351				
	Total	260,871.843	3615					

**Table 3:** Results of normality tests (Shapiro–Wilk and Anderson–Darling) for base frequencies across datasets ($M_{seqs[]}$, $M_{seqs}$, and ***RNAbase***).

Dataset	Base	Shapiro–Wilk (Stat, *p*-Value)	Anderson–Darling (Stat)
$M_{seqs[]}$	U	0.9745, 0.0000	11.6846
	G	0.9752, 0.0000	11.6548
	A	0.9739, 0.0000	12.4270
	C	0.9765, 0.0000	11.6131
$M_{seqs}$	U	0.9811, 0.0000	109.7242
	G	0.9807, 0.0000	116.2862
	A	0.9808, 0.0000	110.7593
	C	0.9803, 0.0000	116.5028
**RNAbase**	U	0.9079, 0.0000	17.4529
	G	0.9171, 0.0000	16.7724
	A	0.9076, 0.0000	22.9033
	C	0.9407, 0.0000	13.0821

**Table 4:** Kolmogorov–Smirnov test for pairwise distributional differences between base frequencies across datasets.

Base	Comparison	D-Statistic	*p*-Value
**U**	$M_{seqs}$ vs. $M_{seqs[]}$	0.4727	~0.0
	$M_{seqs[]}$ vs. ***RNAbase***	0.2215	~0.0
	$M_{seqs[]}$ vs. ***RNAbase***	0.5433	~0.0
**G**	$M_{seqs}$ vs. $M_{seqs[]}$	0.4925	~0.0
	$M_{seqs}$ vs. ***RNAbase***	0.3423	~0.0
	$M_{seqs[]}$ vs. ***RNAbase***	0.6456	~0.0
**A**	$M_{seqs}$ vs. $M_{seqs[]}$	0.4819	~0.0
	$M_{seqs}$ vs. ***RNAbase***	0.2404	~0.0
	$M_{seqs[]}$ vs. ***RNAbase***	0.6352	~0.0
**C**	$M_{seqs}$ vs. $M_{seqs[]}$	0.4786	~0.0
	$M_{seqs[]}$ vs. ***RNAbase***	0.2752	~0.0
	$M_{seqs[]}$ vs. ***RNAbase***	0.6471	~0.0

**Table 5:** The best folded aptamers including sequences, secondary structures, and tertiary structures from the three datasets.

Aptamer ID	Sequence (5′ to 3′) and Pseudoknots	MFE 2d Structure	Tertiary Structure/3D Structure
***RNAbase***			
RNAse69	AUUUCUCUGAGAUGUUCGCAAGCGGGCC AGUCCCCUGAGCCGAUAUUUCAUACCAC AAGAAAUGUGGCGCUCCGCGGUUGGUGA GCAUGCUCGGUCCGUCCGAGAAGCCUUA AAACUGCGACGACACAUUCACCUUGAAC CAAGGGUUCAAGGGUUACAGCCUGCGGC GGCAUCUCGGAGAUUCC ...((((((((((((.(((..((((((.........(((((.(((((((.........))))))). ).))))(((((((..((((....(((((.....)))))....)))).)))))))............(( (((((((...)))))))))......)))))).)))))))))))))))....	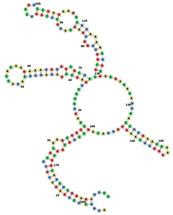	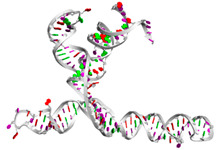
RNAse192	GGGAGAAUUCCGACCAGAAGCUUGUGAG ACCAGCCGAGUGGUGUCUGGCUAUUCAC UGGAGCGUGGGUGGAACCCCUGCGCACU CGUUUGGCUGUCCGGGCCUUCGGGCCGG GAUUAUCUCUUUGGGUUUUGUGAUUUGG UCAUAUGUGCGUCUACAUGGAUCCUCA ((((.(((((((.((.((((((((.((.(((((.((((((.(((..((((((((((......))) )))))...))..))))))))).)))).).)))))).)))).)).))))))).))))........... (((...((((.((((((....)))))))))).)))	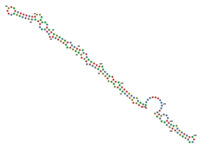	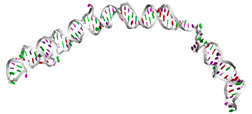
$M_{seqs}$			
aptamerd5165	CAAGCACACCACGAUGCCCCA CGCAUCGUGGUGUGGCACAUC CAGCGUGAGCGA ....(((((((((((((.....)))))))))))))((.(((.....))).))..	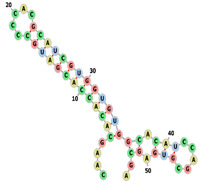	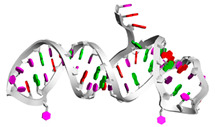
aptamerd18670	UGCCAUUGCUGCCUGUGCUGU GUUGGUUGGAGCGCAGCUAGC AAUGGAGCG ..(((((((((.(((((((.((.....)).))))))).)))))))))....	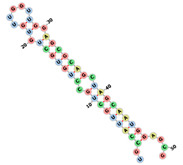	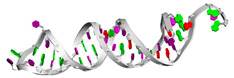
$M_{seqs[]}$			
Aptamer1084	CGUUGGCUUAGUCACUAAGCCA ...((((((((...))))))))	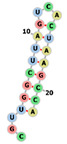	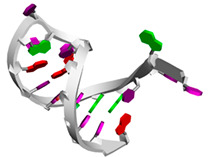
Aptamer960	GGCCCGGACUAGUCAUUCGGGC .(((((((.......)))))))	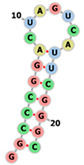	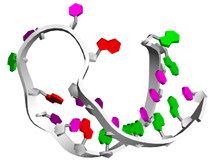

## Data Availability

The supplementary data are provided with the documents. The datasets generated and analyzed during the current study are publicly available on GitHub website at: https://github.com/KPMOKGOPA/Benchmarmking-Datasets accessed on (1 August 2025). This repository includes the randomized aptamer sequence datasets (Mseqs and Mseq[]), along with basic annotations such as sequence length, base composition, and computed MFE values.
